# An affordable and immersive virtual reality-based exercise therapy in forward head posture

**DOI:** 10.1371/journal.pone.0297863

**Published:** 2024-03-06

**Authors:** Afsoon Asadzadeh, Zahra Salahzadeh, Taha Samad-Soltani, Peyman Rezaei-Hachesu

**Affiliations:** 1 Department of Health Information Technology, School of Management and Medical Informatics, Tabriz University of Medical Sciences, Tabriz, Iran; 2 Department of Physical Therapy, Faculty of Rehabilitation Sciences, Tabriz University of Medical Sciences, Tabriz, Iran; UFPE: Universidade Federal de Pernambuco, BRAZIL

## Abstract

Forward Head Posture (FHP) is one of the most commonly occurring musculoskeletal abnormalities. Despite exercise therapy being an effective approach for FHP treatment, it can be long, monotonous, and tedious. Virtual reality (VR) can be used as an innovative solution to address these challenges. We designed an affordable and immersive VR-based exercise therapy (VRET) system for FHP correction. The VRET contents (i.e., exercises and VR scenarios) were determined by physiotherapists and game designers at the focus group meetings. Hardware requirements include a VR box, smartphone, and sensors (i.e., a smartphone accelerometer and an affordable Inertial Measurement Unit (IMU)) to measure head motions and transfer them via Wi-Fi to the VRET system. The IMU was designed using the MPU6050, Arduino Nano, and ESP8266-01S. Gwet’s AC1, Game Experience Questionnaires (GEQ), and System Usability Scale (SUS) were used to measure intra-rater reliability, user experience, and system usability, respectively. The determined exercises, including Capital Flexion-Extension and Chin Tuck, were designed in the form of a shooting game. A physiotherapist and twenty-one FHP individuals took part in evaluating the system. High precision was obtained for the designed IMU (i.e., pitch and roll < 0.1° and yaw < 1.3 °). Gwet’s AC1 and SUS results showed very good intra-rater reliability (coefficient = 0.892) and excellent usability (score = 87.14), respectively. According to the mean scores of the GEQ, participants were confident about competence, immersion, flow, and positive affect components. The development of low-cost VRET systems for FHP correction is a step towards facilitating rehabilitation challenges by providing positive experiences for users as well as helping them perform therapeutic exercises correctly.

## Introduction

### Forward head posture

Ensuring proper posture involves imposing minimal stress on the body through maintaining musculoskeletal balance [1]. Different postural deformities, such as kyphosis, rounded shoulders, scoliosis, lumbar lordosis, and Forward Head Posture (FHP) have been thus far prevalent because of the tendency in individuals to inappropriately carry themselves in a physical manner [2]. FHP, in particular, is considered as one of the most commonly occurring musculoskeletal abnormalities characterized by poor head posture in the sagittal plane and a more anteriorly leaning, rather than a vertical line of the body’s center of gravity with respect to the head position. This condition exerts increased loads on the cervical joints and ligament system and even causes load increments and a kind of biomechanical tension on the cervical spine [1, 3–5]. The prevalence rate of FHP worldwide exceeds 66% and it occurs at various severities, i.e., slight, moderate, or severe [2, 6].

Numerous studies have so far identified a variety of causes and risk factors of FHP, such as recurring and cumulative trauma to the neck, long-time sitting, as well as inappropriate head posture during work, and use of visual display terminals and smartphones while assuming an improper stance [7–9]. Several disorders and conditions related to FHP, including thoracic kyphosis and cervical range of motion (ROM), postural control, muscle weakness and disorder, pain, and injury can lead to neck pain, headaches, and migraines, as well as postural abnormalities [10–17]. The findings supported by clinical study have reported that nearly 60% of individuals with FHP are affected by neck pain [18]. If left untreated, this health problem may even reduce functions of the muscular system and limit cervical mobility [19].

Some typical physical methods of correcting FHP and encouraging patient compliance with treatment plans are thermotherapy, electric stimulation therapy, traction, manual therapy and therapeutic exercises [18, 20]. Exercise therapy is one of the therapeutic methods that has been suggested to treat FHP [21–23]. Sheikhhoseini et al. reported in their systematic review that different exercise programs (e.g., stretching and strengthening exercises of the spine and hamstrings; a home exercise program, including 2 strengthening and 2 stretching exercises for the neck; Pilates exercise, including stretching and strengthening exercises for the neck and upper back; and Rocabado 6 x 6 exercises for the neck and jaw) had an effect on FHP correction [24]. For example, Harman et al. showed that a 10-week exercise program that comprised two strengthening exercises for deep cervical flexors and shoulder retractors and two stretching exercises for cervical extensors and pectoral muscles caused significant improvements in asymptomatic participants of cervical ROM and postural misalignment [25].

However, nonadherence to prescribed exercises, in particular home exercise programs, due to multifactorial reasons, such as self-efficacy, pain, psychological symptoms, physical activity, and social support is a significant problem that can potentially have a detrimental effect on treatment outcomes [26]. Therefore, therapists need a meaningful and motivational tool to increase the effectiveness of exercise therapy. Accordingly, VR, as an innovative technology, can be used to respond to these challenges [27–29].

### Virtual reality

VR is defined as “the use of interactive simulations created with computer hardware and software to present users with opportunities to engage in environments that appear to be and feel similar to real-world objects and events” [30]. Virtual environment, interactivity, immersion environment, and sensory feedback are essential factors of VR [31]. Various information technologies (ITs), such as computer graphics, image processing, pattern recognition, Artificial Intelligence (AI), networking, and audio systems, as well as different hardware and software can be used to design and implement VR systems [31–33]. This technology has been used as an interactive, motivational, and assessment tool in the rehabilitation field [29, 34–37]. The features of VR-based games lead users to forget their problems or disease when immersed [28]. Numerous studies have been conducted to show the capabilities of VRET systems to treat neck pain [38–40]. For instance, Mihajlovic et al. indicated that serious game-based VR makes the users more motivated to continue head-neck rehabilitation exercises [40]. Moreover, Chen et al. asserted that visual feedback of VR could promote the neck movements of asymptomatic individuals and neck pain patients [38].

### Hardware requirements in VR-based neck rehabilitation

A head-mounted display (HMD) (e.g., Oculus Rift and HTC Vive) is a suitable device for measuring neck axis angles and providing immersion features [41]. Motion Capture is also accurate hardware to measure head rotation, but adjusting it for the user is time-consuming [42]. Kinect Xbox360 is another device that is used for neck exercises, but it has insufficient precision for measuring the neck yaw axis [43]. However, it is called affordable, portable, markerless, and a wonderful device for the assessment of gross movement-based impairment [44, 45]. The high cost of the hardware is one of the VR limitations that should be addressed as much as possible by developing economical devices [46–49]. Accordingly, Kim et al. developed a low-cost, high-performance, and accurate infrared optical head tracker for head posture measurement with approximate precision of 4.5° and 8.0° for 1 and 3D positions, respectively [50]. In addition, the use of VR headsets which uses the smartphone as display can be used in affordable VR system [51].

### The rest of the paper

FHP disorder is one of the most common postural disorders that has various side effects in untreated or prolonged individuals. Although exercise therapy is a proven method for FHP treatment, it can has various challenges, such as long duration, monotonous and tedious. VR has increasingly been incorporated into rehabilitation processes for various pathologies and conditions, with the aim of enhancing the results obtained by traditional methods; It also has the potential to address the exercise therapy limitations and facilitate the treatment of FHP. Therefore, the VRET systems can be used as a motivating, assisting, and monitoring solution in the rehabilitation of FHP individuals, especially at home. To our knowledge, no previous study has been conducted on VRET in FHP correction. Therefore, our study aimed to answer the following questions:

1. How can we design an affordable game-based VR for FHP exercise therapy with focus on improving deep neck flexor muscles?
2. Is the VR system designed in this study approved by experienced physiotherapists for use in rehabilitation clinics?
3. What is the user experience of FHP individuals in using the designed virtual reality system?

## Materials and methods

### Ethics statement

This research was approved by the research ethics committee of the Tabriz University of Medical Sciences (Number: IR.TBZMED.REC.1398.299) [52]. We informed the physiotherapists and FHP individuals about the aim of this research and the volunteer subjects gave their written informed consent to take part in this study.

### System design requirements

**Fig 1:**
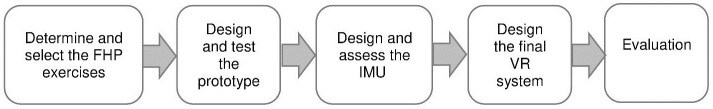
The design process of game-based VR on FHP correction.

As shown in Fig 1, the design and evaluation of our VR-based system for FHP exercise therapy consist of five steps. The details of these steps are explained below.

### Determine and select the FHP exercises

First, a physiotherapist with 20 years of experience in postural corrections recommended specialized corrective exercises for FHP to be designed in the VR system. The criteria for selecting exercises were as follows:

1. **Feasibility in Virtual Reality (VR) Format:** The exercises should be producible in a virtual reality format with immersive features.
2. **Strengthening Deep Neck Flexor Muscles**: The exercises should focus on the deep neck flexor muscles in thos stage.
3. **Commonly Practiced Exercises**: The chosen exercises should be commonly practiced.
4. **Accessibility for Home Practice (HP)**: Individuals should be able to easily perform these exercises at home using a VR product.

Subsequently, two focus group meetings were conducted at the Faculty of Rehabilitation, Tabriz University of Medical Sciences, to validate the selected exercises. The first focus group comprised three physiotherapists with at least 18 years of experience in FHP treatment. The second focus group included 10 physiotherapists with over 10 years of work experience to further validate the chosen exercises.

### Game scenario

In order to explore the viewpoints of the physiotherapists (N = 3), technical teams (N = 5) and users (N = 4) for game scenario, three 90-min focus group discussion (FGD) sessions were held in School of Management and Medical Informatics, in a meeting room.

### Design and test the prototype

This stage investigates the ability of the smartphone accelerometer to provide the interactive features of selected exercises in the VR environment. This step is divided into three levels as follows: (1) determine the game scenario through brainstorming among game designers and physiotherapists (N = 6), (2) design the determined scenario in the Unity software, and (3) test the prototype on a FHP individual (age = 12 years old).

**Fig 2:**
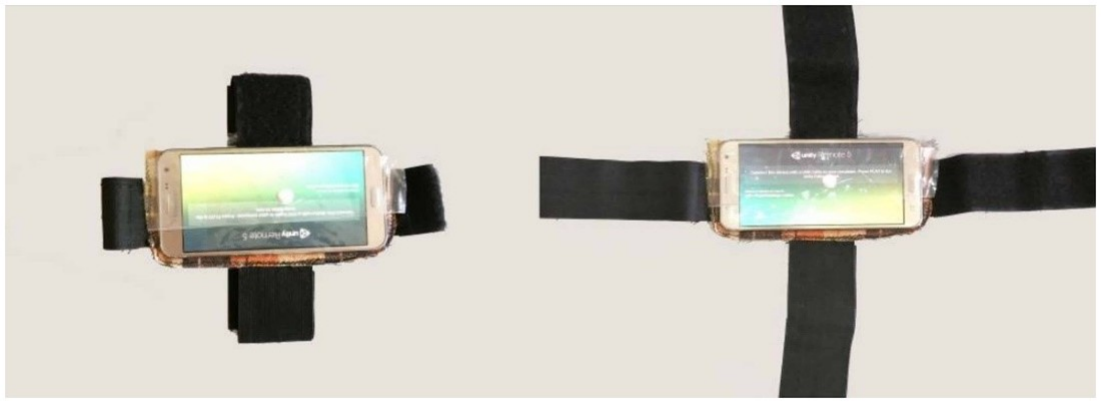
The designed holder to fix the smartphone on the forehead and neck of the person.

In the design step of the prototype, the smartphone accelerometer was activated for measuring pitch and roll. The axes were sent to the Unity software to provide the interactive features of the game prototype using the Formula (1). Finally, we built a holder to fix the smartphone in the head and neck of the FHP sample to test the prototype (see Fig 2).


$$\begin{array}{c} \text{pitch}=Mathf.Atan\left(Input.acceleration.x/Mathf.Sqrt\left(Mathf.Pow\left(Input.acceleration.y,2f\right)+Mathf.Pow\left(Input.acceleration.z,2f\right)\right)\right)*(180/Mathf.PI);\\ \text{roll}=Mathf.Atan\left(Input.acceleration.y/Mathf.Sqrt\left(Mathf.Pow\left(Input.acceleration.x,2f\right)+Mathf.Pow\left(Input.acceleration.z,2f\right)\right)\right)*(180/Mathf.PI); \end{array}$$ (1)

### Develop and evaluate the Inertial Measurement Units (IMUs)

The designed IMU was used for exercises where the smartphone accelerometer couldn’t provide interactive features. Accordingly, we designed three IMUs to measure rotations and send them to the VR environments [49]. We selected affordable hardware for measuring cervical motion (i.e., pitch = cervical lateral flexion, roll = cervical flexion/extension, and yaw = cervical rotation) during exercise therapy. Table 1 shows the hardware and software requirements for the development of the designed IMU.

**Table 1:** Hardware and software requirements for measuring pitch, roll, and yaw.

Hardware	Hardware Price	Software	Programming language
Mpu6050	4$	Arduino IDE	C++ for build-in module
Esp 8266 Esp-01	1.5$	Unity	C# for Unity
Arduino Nano	5$	Altium designer	
USB (Mini-B)	1$		
Breadboard	1$		

The designed IMU comprises three boards: (1) MPU 6050, i.e., a micro-electro-mechanical system with a three-axis gyroscope, three-axis accelerometer, and Digital Motion Processor (DMP), (2) Arduino Nano, i.e., a microcontroller-based on the ATmega, and (3) ESP 8266 ESP-01, i.e., a Wi-Fi module that allows microcontrollers access to a Wi-Fi network [53–56]. A printed circuit board (PCB) was designed with Altium designer software and printed.

### Design the final VR system

VR-BOX (Remax model) andsmartphones with Android 5 or higher have been used in this study. It is low-cost headset that uses a smartphone (model: Samsung J7) as a display like Google Cardboard. The prototype version of the system (see Section 2–3) was improved in this step. We inserted 3D models in the Unity software and arranged them in the form of a farm terrain.

We dedicated the following items to the game menu: (1) Registration, (2) Game introduction, (3) Game instructions, (4) Setting, and (5) Contact with the research team, and Quit. The method of data storage is client-based, meaning that the demographic information of the registration section and the gameplay data are stored on the individual’s mobile phone in the following path: /storage/emulated/0/Android/data/com.Tbzmed.VRET4FHP/files. Physiotherapists can monitor the FHP individuals through stored information, including personal information, frequency of exercise sets, the correct frequency of exercise, and time & date on the application. The designed IMU or smartphone accelerometer transmits neck motion to the game. In other words, to integrate sensors with the VR system or send neck rotations to the VR system, ESP8266 01 transmits the measured angles to the Wi-Fi network using soft access point mode. Then a socket listens on port 80 until it receives a request for neck motion data. In this local network, the IP address 192.168.4.1 belongs to the VR system, which receives the pitch, roll, and yaw angles of the neck while the user performs exercises. If the user performs the exercises correctly, the projectile will hit the target. The users can customize the range of neck motion angles within the application.

### Calibration of the IMU

Before using the VR system by FHP participants an initial calibration was performed and IMU parameters (i.e, biases and sensitivities) was adjusted by developers. To ensure accurate measurement of neck rotation using an IMU (Inertial Measurement Unit), the following steps was educated for users to calibrate IMU:

Wave the IMU in a Figure-of-Eight:

- Hold the IMU and move it in a figure-of-eight pattern for at least 10 seconds.
- Ensure that it rotates through all three axes (x, y, and z) during this motion.

Spin the IMU on a Flat Surface:

- Place the IMU on a flat surface.
- Spin it through two full rotations around the z-axis (vertical axis).
- Avoid placing it near metallic objects to prevent interference.

Wait for Stabilization:

- After spinning, wait at least five seconds for the data to stabilize.

Rotate on the Flat Surface:

- Continue rotating the IMU on the flat surface for at least five seconds until the z angle is also zero.

Attach IMUs to Subjects:

- Once each sensor is calibrated, attach them to your back neck.
- Collect data in the required collection mode for your VR environment.

#### Evaluate the VR system

We evaluated the VR system in two phases:) 1) approving the reliability of the system by the physiotherapist and) 2) system usability and user experience through FHP individuals, which were explained in the following.

*Reliability of system*. An expert physiotherapist in the performing of FHP exercises evaluated the designed VR system within two days. The physiotherapist performed all three levels of the game in two rounds (i.e., 2 sets of 15 replicates). She performed exercises while immersed in the VR system, and reported her score with 0 or 1 (i.e., 0 means false shooting and one means correct), as well as, the technical team checked her feedback and game scores synchronously. The Unity remote software was used to display the game on the laptop and the physiotherapist’s headset.

Descriptive statistics were used to report the percentages of system and physiotherapist scores. Gwet’s AC1 value was used for intra-rater reliability [57]. Gwet’s AC1 is a statistical measure of inter-rater reliability, which means the degree of agreement among two or more raters who assign categorical ratings to the same subjects. Gwet’s AC1 is an alternative to Cohen’s Kappa. The strength of agreement was categorized according to Gwet’s AC1 as follows: < 0.2 = poor; 0.21–0.4 = fair; 0.41–0.6 = moderate; 0.61–0.8 = good; and 0.81–1.0 = very good [58].

*Usability and user experience evaluation*. ***Participants*.** The subjects were determined through convenience sampling based on their availability and ease of access. Recruitment occurred from December 31, 2022, to June 24, 2023, in three local universities (i.e., Tabriz University of Medical Sciences, Tabriz Islamic Art University, and Tabriz University) in Iran. A total of 35 student volunteers from three local universities were screened by measurement of the craniovertebral angle (CVA) through photogrammetry. CVA is one of the popular and more accurate indicators for measuring head posture than the head tilt and the head position angle [4, 16]. In this study, students with CVA<49 degrees identified as having FHP disorder [59], and individuals were excluded if CVA ≥ 50°. The length of the procedure was set at 30 minutes per included subject.

Two modules of the Game Experience Questionnaire (GEQ) with a Likert scale ranging from 0 = not at all to 4 = extremely, including **a)** the in-game questionnaire and **b)** the post-game questionnaire (PGQ) were used to evaluate user experiences [60]. The in-game questionnaire, a concise version of the core questionnaire, consists of 14 questions with seven components, including (1) competence, (2) sensory and imaginative immersion, (3) flow, (4) tension, (5) challenge, (6) negative affect, and (7) positive affect. Each component has three items that are rated on a 5-point Likert scale, ranging from 0 (not at all) to 4 (extremely). The total score for each component is calculated by averaging the scores of the three items. The PGQ is designed to assess the players’ feelings and thoughts after they have finished playing the game. It was used to evaluate the overall satisfaction, enjoyment, and engagement of the players, as well as their emotional and cognitive responses to the game. It comprises 17 questions which are divided into four components, including (1) positive experience, (2) negative experience, (3) tiredness, and (4) returning to reality. Additionally, the System Usability Scale (SUS) was used to measure the system usability. This questionnaire comprises 10 items with a Likert scale ranging from 1 = strongly disagree to 5 = strongly agree. Iزt provides an easy-to-understand score from 0 (negative) to 100 (positive) [61].

## Results

We presented the findings in five sections as follows: 1) content determination, 2) design of the VR system’s prototype, 3) designed IMU, 4) design of the final VR system, and 5) evaluation.

### Content determination

Table 2 shows the exercises that were confirmed at the focus group meetings to develop in the VR form.

**Table 2:** Approved exercises in the focus group session.

Exercises	Description	Variable joints	Exercise Frequency,	Variable axes
Chin tuck	Tuck the chin to the chest, then rotate the head to the left and right	C1-T7	3 sets of 15 repetitions	Roll and yaw
Capital flexion-extension	Sit on a chair, bring slowly the head downward and upward	Atlantooccipital and atlantoaxial joints	3 sets of 15 repetitions	Roll

To strengthen the deep neck flexor muscles, **chin tucks** and **capital flexion and extension** can be used as useful therapeutic exercises. Chin tucks are not only great for strengthening, but also improve both stability and functional strength, while assisting in injury prevention for the neck. The muscles involved in these exercises are as follows:

1. **Flexion Movement**: Longus colli, Sternocleidomastoid, Scalene anterior, Longus capitis, Rectus capitis anterior (head only)
2. **Extension Movement**:Levator Scapulae, Splenius cervicis,Splenius capitis, Trapezius, Erector spinae, Rectus capitis posterior (major and minor) (head only)
3. **Chin Tuck**: Splenius, Semispinalis capitis, Semispinalis cervicis, Longissimus capitis, Longissimus cervicis, Longus capitis, Longus colli, Involved muscles during rotation:

- Semispinalis cervicis
- Multifidus
- Scalene anterior
- Splenius cervicis and capitis
- Sternocleidomastoid
- Inferior oblique (head only)
- Rectus capitis posterior major (head only)

#### Game scenario

The FGD members determined recommendations for the game scenario as follows: 1) anchoring content to reality, 2) no need for hand movements during the game, 3) providing rewards in terms of scores, 4) providing audio feedback, and 5) single user. The idea that was agreed to be implemented in the VR environment is "concentration to achieve the championship". Accordingly, various scenarios were obtained in the brainstorming, but due to the following reasons, the shooting idea was chosen as the game scenario.

- The shooting scenario requires concentration, and the participant can not do it unless the FHP individual concentrates on his/her neck motions.
- This scenario does not require a dynamic environment and the participant can do it while sitting on a chair.
- It can be repeated 15 times without changing the complexity of the game (each exercise should be done 15 times).
- The shooting game is a familiar and interesting scenario for men and women.
- The challenge of the game can be provided according to the movements of the neck (i.e., the complexity of the exercises). This is completely consistent with the clinical aspects of this research.

### Prototype of the VR system

**Fig 3:**
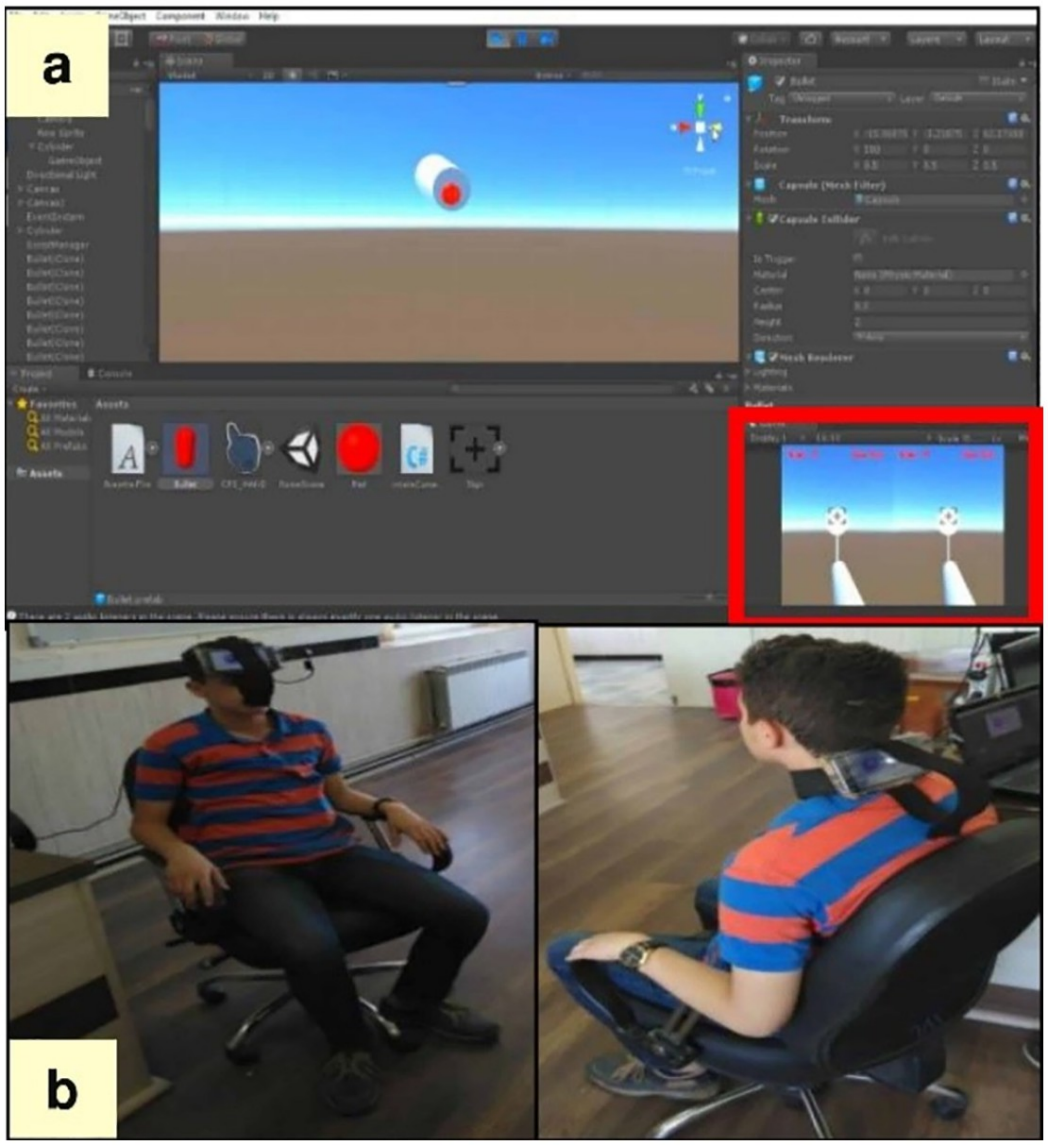
Prototype stage. a The shooting scenario in the prototype stage. b Test of the prototype on the subject with FHP.

Fig 3 illustrates the prototype test in the FHP individual. For Capital Flexion-Extension, the roll axis is variable. When a smartphone is attached to the forehead or placed inside the VR box, the roll axis of the Capital Flexion-Extension exercise can be successfully measured and transferred to the VR environment. The roll and yaw axes are variable in the chin tuck exercise. To measure these axes, the smartphone is required to be placed behind the neck, but we had two challenges for this exercise: the yaw axis of this exercise cannot be measured by the accelerometer, and the smartphone’s gyroscope is needed for it. However, our participants didn’t have gyroscopes on their smartphones. Even if the smartphone’s gyroscope is used for the Chin-Tuck exercise, the head must bear the weight of four pieces of hardware (i.e., two smartphones, a VR box, and a fixed holder for the smartphone), which are harmful to the FHP individuals. In other words, a smartphone with a fixed holder needed to be mounted behind the neck, as well as the headset, and another smartphone must be placed in front of the user’s eyes. Consequently, smartphone sensors are not a suitable solution for the Chin Tuck exercise. The research team needed a light and affordable sensor that could solve our challenge. Therefore, we designed the low-cost IMUs, which are explained in the next section.

### Designed IMUs

**Fig 4:**
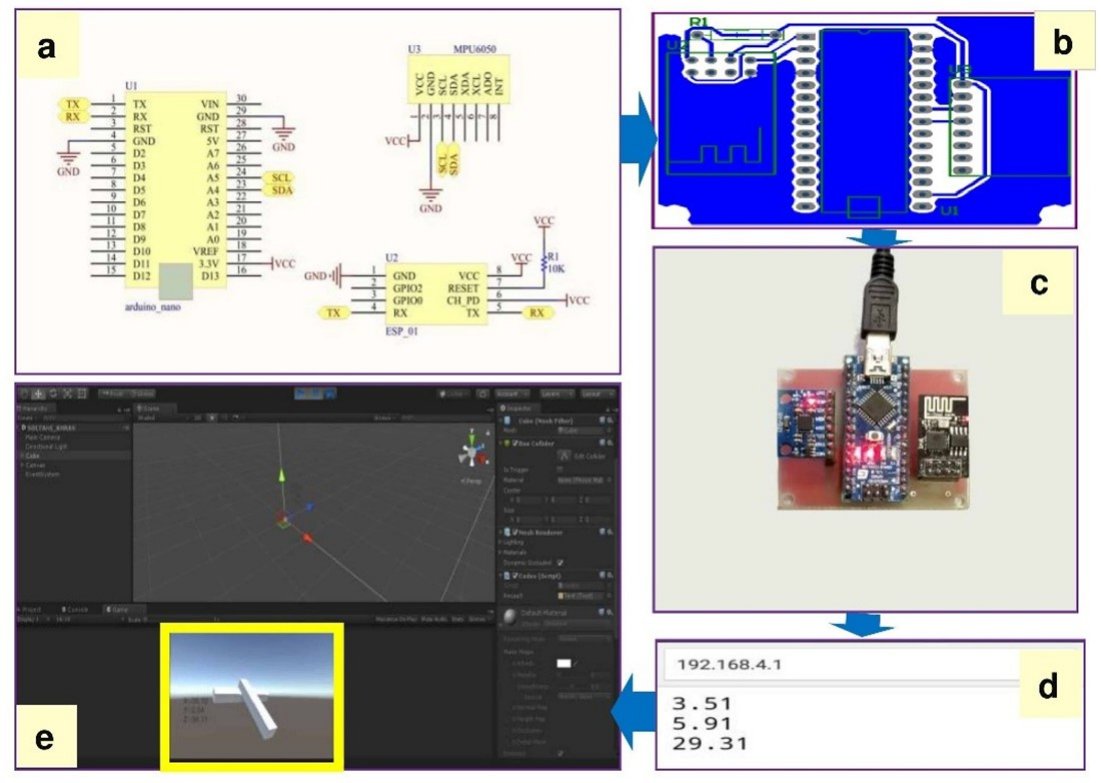
IMU design process. a The schematic output from Altium designer software. b Designed PCB for the designed module. c Show axes on IP: 192.168.4.1. d Chips assembly on PCB. e Pitch and roll outputs in Unity environment.

Fig 4 shows the schematic output of Altium designer software, printed PCB, and transferred motions to the Unity software. The module accuracy is less than 0.1° for the pitch and roll axes, as well as less than 1.3° for the yaw axis.

### The final VR system

**Fig 5:**
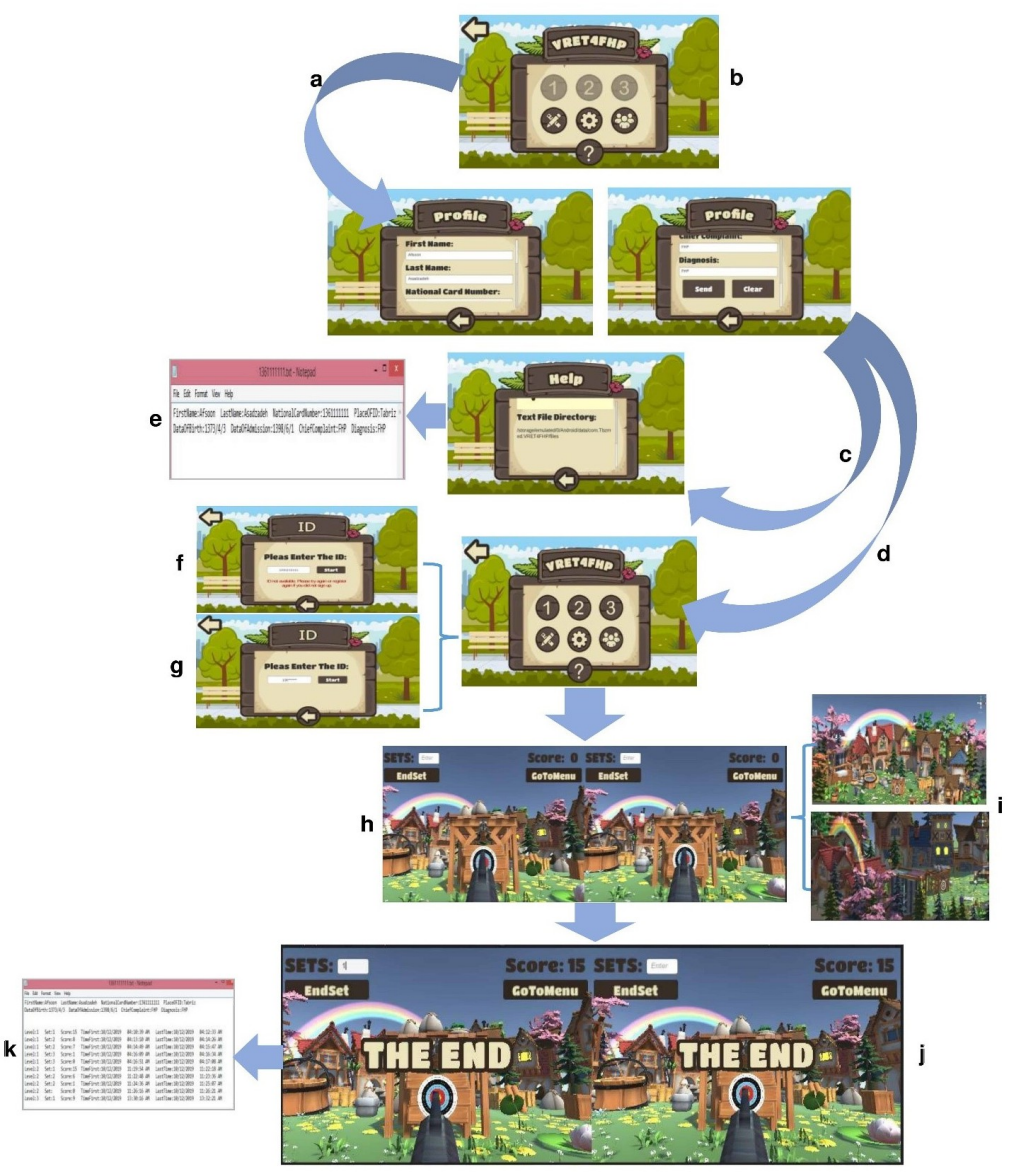
Game-based VR system process. a User registration. b Game menu before playing. c Send data in a defined directory. d Select each level to start the game. e Stored user information. f Enter incorrect password. g Enter the correct password. h Start the game. i Game theme. j The end game. k Stored game information to control and monitor.

**Fig 6:**
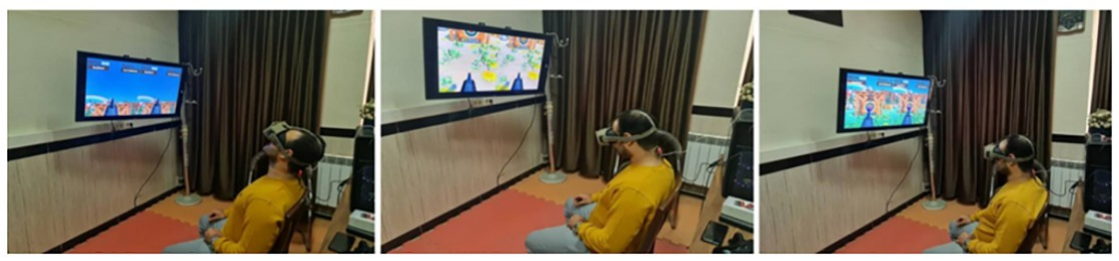
Performing the FHP correction exercise through the designed game-based VR system.

Figs 5 and 6 shows the designed system named VRET4FHP (Virtual Reality-based Exercise Therapy for FHP) and FHP individual during playing the game, respectively. The game menu consists of eight buttons: three buttons for each exercises (i.e., Capital Flexion, Capital Extension, and Chin Tuck) profile, game settings (i.e., sound volume and light), about us, game guidance, and quit. Before starting the game, the player must register their information, including first name, last name, national ID, place of ID, date of birth, chief complaint, and diagnosis. The user information is stored in the defined directory on the smartphone and its path is displayed in the "Help" section of the game. After registering, three levels of exercises are activated in the game. When the user selects each level, the system requests an ID code for authentication. The final system uses the accelerometer in the smartphone that is acting as the display and the designed IMU to measure the neck’s rotations in Capital Flexion/Extension, and Chin Tuck exercises, respectively. In order to play the game, the designed IMU must mount to the participant’s neck, as well as, the smartphone must be put inside the VR box. The items defined in the game scene include the name of the game, game menu, score, sets number, and end set buttons. The FHP person was guided on how to perform the exercises in the VR by the research team. They should perform the exercises while sitting in a stationary chair. When the user put the VR box on the head, the game enters the Ready state where the gun is ready to fire based on neck motions. The user performs 15 repetitions for each exercise, and the system sends score, sound, and visual feedback to inform them about the exercise performance. During the game, players earn a point for each correctly performed exercise. Specifically, if the neck movement angles align with the predefined angular rules for coordinated exercises, shooting occurs at the center of the target, and the player scores a point. Additionally, the sound of clapping is played in the VR environment. However, if players fail to perform the exercises correctly, shooting occurs in other parts of the target or other areas in the virtual reality space, resulting in the player losing points for that shot. Furthermore, a simultaneous sound encourages them to try again, with the announcement: “Please correct the neck range of motion in the next shooting” After successful level completion, the following sounds are played to show the end of the game: “wordless music to end the game” and “you have completed this stage by receiving x points”. The physiotherapists can control and monitor the adherence of the FHP individual through the game results, which will be stored in the data file.

### Evaluation of the final VR system

#### Reliability

As shown in Table 3, on the first and second days of evaluation, the VR-based system gives correct feedback in 93% (42/45) and 91% (41/45) of exercises, respectively. Gwet’s AC1 is 0.892, which indicates the very good reliability of the VR system.

**Table 3:** Physiotherapist scores for the VR system when performing exercises.

Day	Level 1 (Capital Flexion)		Level 2 (Capital Extension)		Level 3 (Chin Tuck)	
	VR system’s total scores	The physiotherapist’s total scores	VR system’s total scores	The physiotherapist’s total scores	VR system’s total scores	The physiotherapist’s total scores
**First day**	15	15	15	13	15	14
**Second day**	15	14	15	13	15	14

#### User experiences and system usability scale

Twenty-one FHP individuals, who averaged 26.8 years of age (range = 22 to 37 years, SD = 3.4), met the study criteria to participate (see Table 4). They performed both exercises while sitting on a chair.

**Table 4:** Overview of participations information.

Data	Percentage
**Gender**	
Male	76%
Female	24%
**Education**	
PhD students	24%
MSc students	62%
BSc students	14%
**University**	
Tabriz university of medical sciences	38%
Tabriz Islamic Art University	14%
Tabriz university	48%
**VR experience**	
No	38%
Only once	19%
Few times	29%
More times	14%
**Craniovertebral angel (< 49°)**	
Mean (SD)	46.33(1.28)

**Fig 7:**
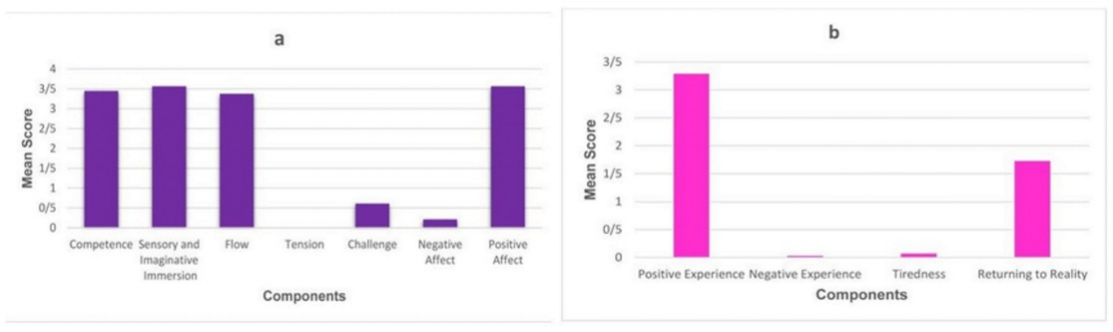
Mean scores of the Game Experience Questionnaire (GEQ) modules. a In-game GEQ. b Post-game module.

We presented the mean scores of the GEQ modules (i.e., in-game and post-game questioners) in Fig 7. The results of the in-game module show a high value for positive affect (M = 3.57, SD = 0.54), sensory and imaginative immersion (M = 3.57, SD = 0.50), competence (M = 3.45, SD = 0.66), and flow (M = 3.38, SD = 0.66), respectively. Additionally, low scores were obtained for the negative affect (M = 0.21, SD = 0.41) and challenges (M = 0.61, SD = 0.98), as well as the tension was not reported at all. In the post-game module, a high value was reported for the positive experience, as well as the return to reality is at average levels (M = 1.7. SD = 1.5) while the level of negative experience (M = 0.03, SD = 0.69) and tiredness (M = 0.07, SD = 0.26) are very low. The mean SUS score was calculated 87.14 (SD = 7.8) which indicates the excellent usability of the VR system. During this study, the participants faced the challenge of attaching the sensor to the back of their neck. To overcome this challenge, they received assistance from the physiotherapist/the authors.

## Discussion

We designed and evaluated a game-based VR with interactive, immersive, and sensory features (i.e., auditory and visual feedback) for FHP disorder. Our designed IMU and smartphone accelerometer were used to transfer neck motion to the VR environment during FHP exercises. A positive gaming experience was reported due to the high values for positive affect, and low scores for negative affect, tiredness, and tension components.

In this study, we focus on exercises (i.e., Chin Tuck and Capital Flexion-Extension) that can strengthen the deep neck flexor muscles in individuals with FHP (). Other studies have also explored these exercises. For instance, Kim et al. investigated the major neck extensor muscles (splenii) and the major flexor muscle through the Chin Tuck exercise. Their analysis suggested that the therapeutic effects of the chin tuck exercise on FHP could be inferred [62]. Similarly, Sureshbabu et al. demonstrated that participants exhibited increased neck flexor muscle activation and improved CVA after performing the chin tuck exercises [63]. Capital flexion-extension is another exercise that strengthens deep neck muscles and improves alignment [64]. Beyond the clinical aspects of exercise, technical considerations also play an important role in exercise selection. In our study, we specifically chose exercises that could be designed in the form of immersive VR experiences.

It should be noted that we found no studies for FHP disorder using VR, but some studies were conducted for VRET in other neck disorder in particular neck pain [38–40, 65]. Tejera et al. showed that VR was not superior to neck exercise treatment in improving pain intensity, flexo-extension and lateral-flexion ROM, temporal summation, neck disability, conditioned pain modulation, left-side pressure pain threshold, pain catastrophizing, fear-avoidance beliefs, or anxiety in patients with non-specific chronic neck pain. Moreover, they revealed that immersive VR was more effective in reducing pain-related fear of movement than exercise treatment [65]. Rezaei et al. conducted a study in order to compare the effect of VR-based therapeutic exercise versus conventional proprioceptive training in individuals with neck pain. Their results indicated that the VR group showed greater improvements than the traditional group in reducing neck pain and motor disabilities [66]. Cetin et al. reported that VR can be used as an effective intervention for improving chronic neck pain [67]. Therefore, VR can be used as an useful tool for neck movement disorders. Our work is similar to the Cluster et al. research, which measured neck motion using the smartphone accelerometer and gyroscope [39]. In their study, the yaw and pitch axes are variable, while the yaw and roll axes change in our project. Our findings showed that the smartphone accelerometer is not a suitable solution for the Chin Tuck exercise. Moreover, even if a smartphone gyroscope is used for the Chin-Tuck exercise, the head must bear the weight of four pieces of hardware, which are harmful to the FHP individual. In other words, a smartphone with a fixed holder needed to be mounted behind the neck, as well as the headset, and another smartphone must be placed in front of the user’s eyes. Therefore, a low-cost and low-weight device is required to measure the yaw axis of Chin Tuck. One of the strengths of this study is the hardware section in which we attempted to provide an affordable interactive VR [49]. It should be noted that the cost is an important and considerable issue, especially in home-based VRET.

The designed mpu6050 module accuracy is less than 0.1 degrees for the pitch and roll and less than 1 degree for the yaw axis. Similar studies have been conducted for the measurement of rotations [68–70]. For example, Tao used MPU6050 and Arduino UNO to simulate motion in the Unity software [70]. In their study, it is unclear how the angles were transferred to the Android platform without the use of a Wi-Fi or Bluetooth chip. There is also no information about the accuracy of the obtained axes. Moreover, Qiu et al. used two JY901 modules and a Bluetooth chip to get knee pitch and roll axes with 0.05 precision, and the yaw axis was not calculated [68]. In another study conducted by Su et al., XBee wireless and 9-DoF RAZOR IMU were used to measure joint angles [69]. They did not report the accuracy of the module and the details of their method to get motion tracking. For developing affordable and immersive VR systems, low-cost alternatives that use smartphones as the display (e.g., Google Cardboard) may be useful. In other words, considering the purchasing power of customers, particularly in low-income or middle-income countries, is very important in designing virtual reality products.

The results of the evaluation showed that the VR system has excellent reliability. However, there are a few errors in the system that are more related to inaccurate calibration, which can be solved by testing, practicing, and user training. A number of studies have used GEQ and SUS to evaluate user experiences and system usability, respectively [71–74]. For instance, Boletsis and Kongsvik examined the Drum-Like VR Keyboard and reported a good usability value due to a mean SUS score of 85.78 (SD: 6.1), as well as, GEQ results demonstrated the interface of the system was easy to use due to low scores in the negative dimensions, i.e., challenge, tension, negative affect and tiredness, and high values for competence [74]. In the present study, the results of the evaluation revealed that the SUS scale gave high usability for the VR system, in other words, the use of the system is easy for users. Additionally, the In-game and the Post-game outcomes showed that participants were positive about the game-based VR experience due to low scores of negative dimensions. Low values for challenges indicated that participants did not face many challenges in using the produced product, and they did not have to put a lot of effort into it. Moreover, our system approximately is not boring due to a low score of negative affects. For the immersion feature of our system, the majority of participants reported that they were interested in the story of the game and found it impressive. After the game, the users have positive experiences due to gaining positive feelings, such as victory, satisfaction, energy, power, and pride. According to the participants’ opinions, the return to reality was at an average level. Overall, the built system can provide a positive experience and cause minimal comfort and tiredness.

## Conclusion

In this paper, we presented VRET4FHP, a novel game-based VR equipped with a designed device for FHP correction. This system can be used as a facilitator tool for FHP individuals to perform their therapeutic exercises correctly. Moreover, physiotherapists can monitor user activity through this system. Considering customer purchasing power is a fundamental principle in the development of VRET products, particularly home-based ones. Our IMU can be used as a valuable economical device to measure gestures in VR projects. Experimental studies are recommended to report the effectiveness of this system on satisfaction, motivation, compliance, and treatment of individuals with FHP disorder, as well as on the satisfaction of physiotherapists. It seems that the game-based VR is a useful tool in rehabilitation; therefore, researchers should consider developing the VRET to demonstrate its positive potential in terms of improving care continuity, adherence to exercises, and overall effectiveness.

## References

[1] Lee K-J, Han H-Y, Cheon S-H, Park S-H, Yong M-S. The effect of forward head posture on muscle activity during neck protraction and retraction. *J Phys Ther Sci*. 2015;27(3):977–9. doi: 10.1589/jpts.27.977 25931773 PMC4395757

[2] Griegel-Morris P, Larson K, Mueller-Klaus K, Oatis CA. Incidence of common postural abnormalities in the cervical, shoulder, and thoracic regions and their association with pain in two age groups of healthy subjects. *Phys Ther*. 1992;72(6):425–31. doi: 10.1093/ptj/72.6.425 1589462

[3] Richards KV, Beales DJ, Smith AJ, O’Sullivan PB, Straker LM. Neck posture clusters and their association with biopsychosocial factors and neck pain in Australian adolescents. *Phys Ther*. 2016;96(10):1576–87. doi: 10.2522/ptj.20150660 27174256

[4] Salahzadeh Z, Maroufi N, Ahmadi A, Behtash H, Razmjoo A, Gohari M, et al. Assessment of forward head posture in females: observational and photogrammetry methods. J Back Musculoskelet Rehabil. 2014;27(2):131–9. doi: 10.3233/BMR-130426 23963268

[5] Kim EK, Kim SG. Forward head posture (FHP) angle and plantar pressure resulting from oscillatory stimulation training of the shoulder joint: A randomized controlled trial. J Back Musculoskelet Rehabil. 2019;32(1):37–42. doi: 10.3233/BMR-160748 30056409

[6] Shaghayegh Fard B, Ahmadi A, Maroufi N, Sarrafzadeh J. Evaluation of forward head posture in sitting and standing positions. European spine journal: official publication of the European Spine Society, the European Spinal Deformity Society, and the European Section of the Cervical Spine Research Society. 2016;25(11):3577–82. doi: 10.1007/s00586-015-4254-x 26476717

[7] Kim S-Y, Koo S-J. Effect of duration of smartphone use on muscle fatigue and pain caused by forward head posture in adults. *J Phys Ther Sci*. 2016;28(6):1669–72. doi: 10.1589/jpts.28.1669 27390391 PMC4932032

[8] Lee M-Y, Lee H-Y, Yong M-S. Characteristics of cervical position sense in subjects with forward head posture. *J Phys Ther Sci*. 2014;26(11):1741–3. doi: 10.1589/jpts.26.1741 25435690 PMC4242945

[9] Nejati P, Lotfian S, Moezy A, Nejati M. The study of correlation between forward head posture and neck pain in Iranian office workers. *Int J Occup Environ Health*. 2015;28(2). doi: 10.13075/ijomeh.1896.00352 26182924

[10] Mahmoud NF, Hassan KA, Abdelmajeed SF, Moustafa IM, Silva AG. The Relationship Between Forward Head Posture and Neck Pain: a Systematic Review and Meta-Analysis. Current reviews in musculoskeletal medicine. 2019;12(4):562–77. doi: 10.1007/s12178-019-09594-y 31773477 PMC6942109

[11] Lin G, Zhao X, Wang W, Wilkinson T. The relationship between forward head posture, postural control and gait: A systematic review. Gait & posture. 2022;98:316–29. doi: 10.1016/j.gaitpost.2022.10.008 36274469

[12] Bae W-S, Lee H-O, Shin J-W, Lee K-C. The effect of middle and lower trapezius strength exercises and levator scapulae and upper trapezius stretching exercises in upper crossed syndrome. *J Phys Ther Sci*. 2016;28(5):1636–9. doi: 10.1589/jpts.28.1636 27313388 PMC4905927

[13] Haughie LJ, Fiebert IM, Roach KE. Relationship of forward head posture and cervical backward bending to neck pain. J Man Manip Ther. 1995;3(3):91–7. doi: 10.1179/jmt.1995.3.3.91

[14] Im B, Kim Y, Chung Y, Hwang S. Effects of scapular stabilization exercise on neck posture and muscle activation in individuals with neck pain and forward head posture. *J Phys Ther Sci*. 2015;28(3):951–5. doi: 10.1589/jpts.28.951 27134391 PMC4842472

[15] Lee M-H, Park S-J, Kim J-S. Effects of neck exercise on high-school students’ neck–shoulder posture. *J Phys Ther Sci*. 2013;25(5):571–4. doi: 10.1589/jpts.25.571 24259804 PMC3804985

[16] Yip CHT, Chiu TTW, Poon ATK. The relationship between head posture and severity and disability of patients with neck pain. *Man Ther*. 2008;13(2):148–54. doi: 10.1016/j.math.2006.11.002 17368075

[17] Fernández-de-Las-Peñas C, Cuadrado M, Pareja J. Myofascial trigger points, neck mobility and forward head posture in unilateral migraine. *Cephalalgia*. 2006;26(9):1061–70. doi: 10.1111/j.1468-2982.2006.01162.x 16919056

[18] Ahn J-A, Kim J-H, Bendik AL, Shin J-Y. Effects of stabilization exercises with a Swiss ball on neck-shoulder pain and mobility of adults with prolonged exposure to VDTs. J Phys Ther Sci. 2015;27(4):981–4. doi: 10.1589/jpts.27.981 25995537 PMC4434028

[19] Kang DY. Deep cervical flexor training with a pressure biofeedback unit is an effective method for maintaining neck mobility and muscular endurance in college students with forward head posture. J Phys Ther Sci. 2015;27(10):3207–10. doi: 10.1589/jpts.27.3207 26644676 PMC4668167

[20] Thoomes EJ. Effectiveness of manual therapy for cervical radiculopathy, a review. Chiropractic & manual therapies. 2016;24:45. doi: 10.1186/s12998-016-0126-7 27980724 PMC5146882

[21] Lee SM, Lee CH, O’Sullivan D, Jung JH, Park JJ. Clinical effectiveness of a Pilates treatment for forward head posture. J Phys Ther Sci. 2016;28(7):2009–13. doi: 10.1589/jpts.28.2009 27512253 PMC4968495

[22] Diab AA, Moustafa IM. The efficacy of forward head correction on nerve root function and pain in cervical spondylotic radiculopathy: a randomized trial. Clinical rehabilitation. 2012;26(4):351–61. doi: 10.1177/0269215511419536 21937526

[23] Ruivo RM, Pezarat-Correia P, Carita AI. Effects of a Resistance and Stretching Training Program on Forward Head and Protracted Shoulder Posture in Adolescents. Journal of manipulative and physiological therapeutics. 2017;40(1):1–10. doi: 10.1016/j.jmpt.2016.10.005 27842938

[24] Sheikhhoseini R, Shahrbanian S, Sayyadi P, O’Sullivan K. Effectiveness of Therapeutic Exercise on Forward Head Posture: A Systematic Review and Meta-analysis. Journal of manipulative and physiological therapeutics. 2018;41(6):530–9. doi: 10.1016/j.jmpt.2018.02.002 30107937

[25] Harman K, Hubley-Kozey CL, Butler H. Effectiveness of an exercise program to improve forward head posture in normal adults: a randomized, controlled 10-week trial. *J Man Manip Ther*. 2005;13(3):163–76. doi: 10.1179/106698105790824888

[26] Argent R, Daly A, Caulfield B. Patient Involvement With Home-Based Exercise Programs: Can Connected Health Interventions Influence Adherence? JMIR mHealth and uHealth. 2018;6(3):e47. doi: 10.2196/mhealth.8518 29496655 PMC5856927

[27] Kim J, Son J, Ko N, Yoon B. Unsupervised virtual reality-based exercise program improves hip muscle strength and balance control in older adults: a pilot study. *Arch Phys Med Rehabil*. 2013;94(5):937–43. doi: 10.1016/j.apmr.2012.12.010 23262158

[28] Metsis V, Jangyodsuk P, Athitsos V, Iversen M, Makedon F. Computer aided rehabilitation for patients with rheumatoid arthritis. 2013 international conference on computing, networking and communications IEEE; 2013. p. 97–102.

[29] Weiss PL, Rand D, Katz N, Kizony R. Video capture virtual reality as a flexible and effective rehabilitation tool. *J Neuroeng Rehabil*. 2004;1(1):12. doi: 10.1186/1743-0003-1-12 15679949 PMC546410

[30] Weiss PL, Kizony R, Feintuch U, Katz N. Virtual reality in neurorehabilitation. Textbook of neural repair and rehabilitation. 512006. p. 182–97.

[31] Himma KE, Tavani HT. The handbook of information and computer ethics: John Wiley & Sons; 2008.

[32] Hwang J, Jung J, Kim GJ. Hand-held virtual reality: a feasibility study. Proceedings of the ACM symposium on Virtual reality software and technology2006. p. 356–63.

[33] Pirini M, Bisi MC, Turolla A, Agostini M, Vidale D, Fiorentin A, et al. Postural Rehabilitation Within the VRRS (Virtual Reality Rehabilitation System) Environment. Advanced Technologies for the Rehabilitation of Gait and Balance Disorders: Springer; 2018. p. 335–55.

[34] Pensieri C, Pennacchini M. Overview: virtual reality in medicine. *J Virtual Worlds Res*. 2014;7(1). doi: 10.4101/jvwr.v7i1.6364

[35] Kizony R, Katz N, Weiss PL. Adapting an immersive virtual reality system for rehabilitation. *Comput Animat Virtual Worlds*. 2003;14(5):261–8. doi: 10.1002/vis.323

[36] Powell V, Powell W. Therapy-led design of home-based virtual rehabilitation. 2015 IEEE 1st Workshop on Everyday Virtual Reality: IEEE; 2015. p. 11–4.

[37] Ma H. Research on rehabilitation exercise based on virtual reality technology. Proceedings of the 10th Conference on Man-Machine-Environment System Engineering2010. p. 331–4.

[38] Chen KB, Sesto ME, Ponto K, Leonard J, Mason A, Vanderheiden G, et al. Use of virtual reality feedback for patients with chronic neck pain and kinesiophobia. *IEEE Trans Neural Syst Rehabil Eng*. 2016;25(8):1240–8. doi: 10.1109/TNSRE.2016.2621886 28113774

[39] Kloster M, Babic A. Mobile VR-Application for Neck Exercises. *Stud Health Technol Inform*. 2019;262:206–9. doi: 10.3233/SHTI190054 31349303

[40] Mihajlovic Z, Popovic S, Brkic K, Cosic K. A system for head-neck rehabilitation exercises based on serious gaming and virtual reality. *Multimed Tools Appl*. 2018;77(15):19113–37. doi: 10.1007/s11042-017-5328-z

[41] Xu X, Chen KB, Lin J-H, Radwin RG. The accuracy of the Oculus Rift virtual reality head-mounted display during cervical spine mobility measurement. *J Biomech*. 2015;48(4):721–4. doi: 10.1016/j.jbiomech.2015.01.005 25636855

[42] Branson B, Abnos R, Simmer-Beck M, King G, Siddicky S. Using motion capture technology to measure the effects of magnification loupes on dental operator posture: A pilot study. *Work*. 2018;59(1):131–9. doi: 10.3233/WOR-172681 29355132

[43] Beacon JF, Comeau G, Payeur P, Russell D. Assessing the suitability of Kinect for measuring the impact of a week-long Feldenkrais method workshop on pianists’ posture and movement. *JMTE*. 2017;10(1):51–72. doi: 10.1386/jmte.10.1.51_1

[44] Fern’ndez-Baena A, Susín A, Lligadas X. Biomechanical validation of upper-body and lower-body joint movements of kinect motion capture data for rehabilitation treatments. *2012 fourth international conference on intelligent networking and collaborative systems*: IEEE; 2012. p. 656–61.

[45] Webster D, Celik O. Experimental evaluation of Microsoft Kinect’s accuracy and capture rate for stroke rehabilitation applications. 2014 IEEE Haptics Symposium IEEE; 2014. p. 455–60.

[46] Burdea GC. Virtual rehabilitation–benefits and challenges. *Methods Inf Med*. 2003;42(05):519–23. doi: 10.1055/s-0038-1634378 14654886

[47] Belleman RG, Stolk B, de Vries R. Immersive virtual reality on commodity hardware. Proceedings of the 7th annual conference of the Advanced School for Computing and Imaging2001. p. 297–304.

[48] Checa D, Bustillo A. A review of immersive virtual reality serious games to enhance learning and training. *Multimed Tools Appl*. 2019:1–27. doi: 10.1007/s11042-019-08348-9

[49] Asadzadeh A, Samad-Soltani T, Rezaei-Hachesu P, Salahzadeh Z, editors. Low-Cost Interactive Device for Virtual Reality. 2020 6th International Conference on Web Research (ICWR); 2020: IEEE.

[50] Kim J, Nam KW, Jang IG, Yang HK, Kim KG, Hwang J-M. Nintendo Wii remote controllers for head posture measurement: accuracy, validity, and reliability of the infrared optical head tracker. *Invest Ophthalmol Vis Sci*. 2012;53(3):1388–96. doi: 10.1167/iovs.11-8329 22297495

[51] Kesselman M. Current CITE-ings from the popular and trade computing literature: Google Cardboard–virtual reality for everyone. Library Hi Tech News. 2016.

[52] Research ethics certificate Tabriz University of Medical Sciences; 2019 [https://ethics.research.ac.ir/ProposalCertificateEn.php?id=69413&Print=true&NoPrintHeader=true&NoPrintFooter=true&NoPrintPageBorder=true&LetterPrint=true.

[53] Maybeck PS. The Kalman filter: An introduction to concepts. Autonomous robot vehicles: Springer; 1990. p. 194–204.

[54] Faragher R. Understanding the basis of the kalman filter via a simple and intuitive derivation [lecture notes]. *IEEE Signal Process Mag*. 2012;29(5):128–32. doi: 10.1109/MSP.2012.2203621

[55] Min HG, Jeung ET. Complementary filter design for angle estimation using mems accelerometer and gyroscope: Department of Control and Instrumentation, Changwon National University, Changwon, Korea; 2015.

[56] Zhang L, Sidoti D, Bienkowski A, Pattipati KR, Bar-Shalom Y, Kleinman DL. On the Identification of Noise Covariances and Adaptive Kalman Filtering: A New Look at a 50 Year-old Problem. *IEEE Access*. 2020:1-. doi: 10.1109/ACCESS.2020.2982407 34868797 PMC8638515

[57] Gwet K. Handbook of inter-rater reliability: How to estimate the level of agreement between two or multiple raters. Gaithersburg, MD: STATAXIS Publishing Company. 2001.

[58] Dettori JR, Norvell DC. Kappa and Beyond: Is There Agreement? Global spine journal. 2020;10(4):499–501. doi: 10.1177/2192568220911648 32435572 PMC7222679

[59] Ha SY, Sung YH. A temporary forward head posture decreases function of cervical proprioception. JER. 2020;16(2):168–74. doi: 10.12965/jer.2040106.053 32509702 PMC7248444

[60] IJsselsteijn WA, De Kort YA, Poels K. The game experience questionnaire. 2013.

[61] System Usability Scale (SUS) [https://www.usability.gov/how-to-and-tools/methods/system-usability-scale.html.

[62] Han JW, Kim KH, Bae TS, Blaikie K. Biomechanical Analysis of Chin Tuck Exercise with a Subject-Specific Neck Model for the Forward Headed. International Journal of Precision Engineering and Manufacturing. 2018;19(4):587–92. doi: 10.1007/s12541-018-0071-6

[63] Nishanth H, Aishwarya A. Immediate Effect of Chin Tuck Exercises on Craniovertebral Angle and Shoulder Angle Among Collegiates with Forward Head Posture. Biomedical and Pharmacology Journal. 2021;14(4):2295–8.

[64] Poursadegh M, Azghani MR, Chakeri Z, Okhravi SM, Salahzadeh Z. Postures of the Head, Upper, and Lower Neck in Forward Head Posture: Static and Quasi-static Analyses. Middle East Journal of Rehabilitation and Health Studies. 2023;10(4).

[65] Tejera DM, Beltran-Alacreu H, Cano-de-la-Cuerda R, Leon Hernández JV, Martín-Pintado-Zugasti A, Calvo-Lobo C, et al. Effects of Virtual Reality versus Exercise on Pain, Functional, Somatosensory and Psychosocial Outcomes in Patients with Non-specific Chronic Neck Pain: A Randomized Clinical Trial. International journal of environmental research and public health. 2020;17(16). doi: 10.3390/ijerph17165950 32824394 PMC7460130

[66] I R, M R, S E, S K, A RZ. A Novel Virtual Reality Technique (Cervigame^®^) Compared to Conventional Proprioceptive Training to Treat Neck Pain: A Randomized Controlled Trial. Journal of biomedical physics & engineering. 2019;9(3):355–66. doi: 10.31661/jbpe.v0i0.556 31341881 PMC6613157

[67] Cetin H, Kose N, Oge HK. Virtual reality and motor control exercises to treat chronic neck pain: A randomized controlled trial. Musculoskeletal science & practice. 2022;62:102636. doi: 10.1016/j.msksp.2022.102636 35952621

[68] Qiu Y, Li KM, Neoh EC, Zhang H, Khaw XY, Fan X, et al. Fun-Knee™: A novel smart knee sleeve for Total-Knee-Replacement rehabilitation with gamification. 2017 IEEE 5th international conference on serious games and applications for health IEEE; 2017. p. 1–8.

[69] Su W-C, Yeh S-C, Lee S-H, Huang H-C. A virtual reality lower-back pain rehabilitation approach: system design and user acceptance analysis. International Conference on Universal Access in Human-Computer Interaction: Springer; 2015. p. 374–82.

[70] Tao H. Building Custom Real-Time Sensors for Virtual Reality Applications: UC Merced; 2017.

[71] Schild J, LaViola J, Masuch M, editors. Understanding user experience in stereoscopic 3D games. Proceedings of the SIGCHI Conference on human factors in computing systems; 2012. doi: 10.1080/15213269.2011.620538

[72] Cedergren JE. Evaluating the User Experience and Usability of Virtual Reality Locomotion Techniques: An Empirical Comparison: University of Oslo; 2018.

[73] Hookham G, Nesbitt K, Kay-Lambkin F, editors. Comparing usability and engagement between a serious game and a traditional online program. Proceedings of the Australasian Computer Science Week Multiconference; 2016. doi: 10.1145/2843043.2843365

[74] Boletsis C, Kongsvik S. Text input in virtual reality: A preliminary evaluation of the drum-like vr keyboard. Technologies. 2019;7(2):31. doi: 10.3390/technologies7020031

